# Functional brain imaging using near-infrared spectroscopy during actual driving on an expressway

**DOI:** 10.3389/fnhum.2013.00882

**Published:** 2013-12-24

**Authors:** Kayoko Yoshino, Noriyuki Oka, Kouji Yamamoto, Hideki Takahashi, Toshinori Kato

**Affiliations:** ^1^Department of Brain Environmental Research, KatoBrain Co. Ltd.Tokyo, Japan; ^2^Department of Environment/Engineering, Tokyo Branch, Central Nippon Expressway Co. Ltd.Tokyo, Japan; ^3^Department of Environment/Engineering, Central Nippon Expressway Co. Ltd.Nagoya, Japan

**Keywords:** fNIRS, driving, frontal eye field, outdoor brain activation, acceleration, deceleration, constant velocity driving, U-turn

## Abstract

The prefrontal cortex is considered to have a significant effect on driving behavior, but little is known about prefrontal cortex function in actual road driving. Driving simulation experiments are not the same, because the subject is in a stationary state, and the results may be different. Functional near-infrared spectroscopy (fNIRS) is advantageous in that it can measure cerebral hemodynamic responses in a person driving an actual vehicle. We mounted fNIRS equipment in a vehicle to evaluate brain functions related to various actual driving operations while the subjects drove on a section of an expressway that was not yet open to the public. Measurements were recorded while parked, and during acceleration, constant velocity driving (CVD), deceleration, and U-turns, in the daytime and at night. Changes in cerebral oxygen exchange (ΔCOE) and cerebral blood volume were calculated and imaged for each part of the task. Responses from the prefrontal cortex and the parietal cortex were highly reproducible in the daytime and nighttime experiments. Significant increases in ΔCOE were observed in the frontal eye field (FEF), which has not been mentioned much in previous simulation experiments. In particular, significant activation was detected during acceleration in the right FEF, and during deceleration in the left FEF. Weaker responses during CVD suggest that FEF function was increased during changes in vehicle speed. As the FEF contributes to control of eye movement in three-dimensional space, FEF activation may be important in actual road driving. fNIRS is a powerful technique for investigating brain activation outdoors, and it proved to be sufficiently robust for use in an actual highway driving experiment in the field of intelligent transport systems (ITS).

## Introduction

Driving a vehicle requires use of the higher brain functions such as planning, decision-making, and visual attention, for basic driving operations as well as driving safety. It has been suggested that white matter lacunar infarcts in the frontal lobe might be a predictor of traffic crashes (Park et al., 2013). The function of the prefrontal cortex is thus considered to be significant in driving behavior. The “Global status report on road safety” prepared by the WHO (2009) reported that the number of traffic fatalities has remained constant or declined slightly in the developed countries, but it has increased in most countries. According to a public report in Japan (National Public Safety Commission and National Police Agency, 2013), the number of traffic accidents and resulting injuries has declined in the past few years on ordinary roads, but it has increased on expressways.

Under these circumstances, from the point of view of organizations responsible for highway construction, lighting, signage, and the like, one goal of a study of this kind is the potential development of an evaluation system capable of examining physiologically the effects on the brain of highway design, and identifying ways to improve the ease of driving and highway safety. This requires a technique capable of obtaining information that is potentially useful for improving traffic safety by imaging brain activation while the subject is driving a vehicle. In the field of neuroscience, however, which has been developed through experiments indoors, there has been little development of techniques suited for outdoor activities, where the subjects engage in dynamic activities such as driving a vehicle. Driving simulation experiments have therefore been used to image brain activation related to car driving. The involvement of the prefrontal cortex in driving behavior has been reported in functional magnetic resonance imaging (fMRI) studies (Graydon et al., 2004; Horikawa et al., 2005; Calhoun and Pearlson, 2012). However, these experiments were performed in conditions that differed from actual driving, such as pushing buttons (Graydon et al., 2004) and operating a joystick (Horikawa et al., 2005). Technical problems remained in experiments where the simulation environment included a pedal and a steering wheel (Calhoun and Pearlson, 2012; Schweizer et al., 2013); namely, the human subjects were in a supine position, while the fields of view and depth from the driver's seat were smaller than during actual driving.

Functional near-infrared spectroscopy (fNIRS) has attracted attention in this field, and driving simulation experiments have been conducted using fNIRS with the subjects in a sitting position (Yanaginuma et al., 2007; Watanabe et al., 2011; Tsunashima et al., 2012). However, it is difficult to reproduce the various effects on the body such as gravity, and it is still unclear how the brain functions while driving on an actual road. Compared with an ordinary road, an expressway provides fewer changes in the background, because of the unobstructed view. However, driving speeds are much greater on an expressway and the speed differentials during acceleration or deceleration are higher than those on ordinary roads. In simulation experiments, the subjects are in a stationary state, and there have been no reports on brain activation when a subject is actually driving on an expressway. Accordingly, we conducted this first study to detect the brain activation of drivers as they drive on an actual expressway. Vehicle-mounted fNIRS equipment was used to obtain measurements, and brain activation was imaged during various driving operations, with the goal of acquiring basic data during actual highway driving. After recording the brain activation during operation by normal adult drivers in daytime and nighttime conditions, we investigated the sites of the brain involved in highway driving, that may be relevant in the development of traffic safety measures.

## Methods

### Subjects

Twelve healthy adults participated in this study (eight males and four females, average age: 33.3 ± 4.5 years). Using the Edinburgh Handedness Inventory we confirmed that all the subjects were right-handed. The subjects had no history of mental illness or central nervous disorders, and took no medications on the day of the experiment. Written consent was obtained from the participants before enrollment in the study and the protocol was approved in advance by the ethics committee at KatoBrain Co., Ltd. To ensure the safety of the experiments, the subjects reported their physical condition and the amount of sleep they had the night before the experiment. There were no reports of extreme lack of sleep, and the experiments were conducted after the subjects confirmed that they felt fine. The subjects' average length of driving experience was 11.8 ± 5.8 years. Their frequency of driving was 6.1 ± 1.6 times/week, and their frequency of expressway driving was 4.5 ± 6.5 times/month. Only two subjects had experienced an accident (neither accident involved another vehicle or any personal injuries), and the average number of accidents was 0.2 ± 0.4. The average number of traffic violations was 1.3 ± 1.3 times, mostly for speeding. Since recruitment of the subjects was based on the conditions of age, right-handedness, and frequency of driving on a daily basis, the subjects' genders, their driving histories, and their histories of violations and accidents were completely random.

### Location of the experiment

**Figure 1:**
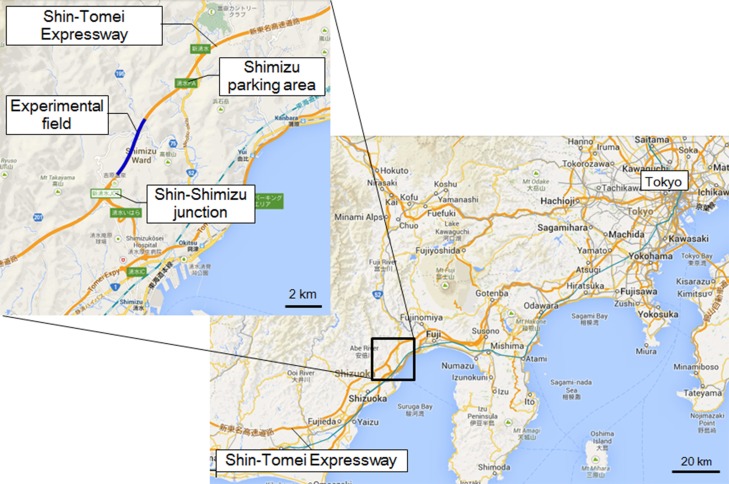
**Location of the experiment**. Location of the Shin Tomei Expressway, showing the experimental course.

The experiment was performed in the Okitsu district, Shizuoka Prefecture, Japan, on a section of the Shin Tomei Expressway immediately before it entered service (Figure 1) (Yamamoto et al., 2012; Kato et al., 2013). The installation of wall parapets, pavement sections, and tunnel lighting had already been completed, so there were no problems with the safety of vehicle travel. The experimental course was restricted to the passage of one vehicle, so there were no vehicles present other than the test vehicle. Guard personnel were stationed at each point on the experimental course and they monitored the status of the road. They could immediately contact the test car using a transceiver if any danger arose on the course, such as an animal intrusion.

**Figure 2:**
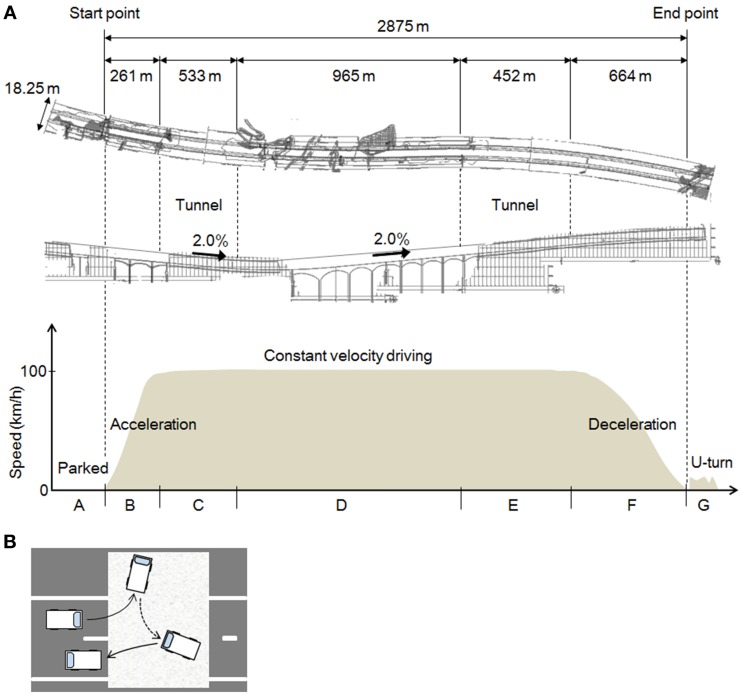
**The experimental field and the tasks. (A)** The experimental task. The shape of the road as viewed from above; a longitudinal view; and a graph of the driving task and vehicle speeds in each section of the course. **(B)** The U-turn task. A tarpaulin was laid down on the road and the subjects were instructed drive on the tarpaulin during the U-turn.

As Figure 2A shows, the full length of the test course was 2875 m, and it included a left curve (*R* = 5000) and a right curve (*R* = 5000). The curves were moderate, so the subjects did not need to engage in difficult handling operations while driving. The slope of the course included a downhill gradient of 2.0% and an uphill gradient of 2.0%, gradual slopes that the drivers hardly noticed. The road width was 18.25 m and there were two lanes (3.75 m wide) on each side. The test vehicle traveled in the left lane, because traffic moves on the left in Japan.

### Experimental procedures

For each subject, the experiment took place over 2 days for preliminary trials and 2 days for the actual experiment. The daytime and nighttime trials were performed on different days for each subject, and the order of the experiments (day or night) was randomized. In the preliminary trials, performed within 10 days of the actual experiment, the subjects drove under the same conditions as in the actual experiment except for the actual course, in the test vehicle, wearing the fNIRS probe attachment. By the time of the actual experiments, the subjects were used to the experimental environment, and none of the subjects complained of any stress.

Diagrams of the different parts of the task are shown in Figure 2A. The task comprised 7 parts (A–G), and one trial consisted of this series A–G. The subjects performed three trials each, in the daytime and at night, on different days.

In A, the test car remained stationary for about 25–30 s in the parking mode of the automatic transmission, with the engine started and the parking brake set. The subject remained in a resting state in the driver's seat. In B, which measured 261 m, the subject accelerated to 100 km/h. The automatic transmission and the parking brake were operated by the experimenter, who rode in the passenger seat. A traffic cone was placed at the end point of B. In C, constant velocity driving (CVD) was maintained at 100 km/h inside a tunnel. After around 5 s at the beginning of C, while the subject adjusted to the feel of 100 km/h, the speedometer was hidden to prevent excessive attention to speed. To ensure the safety of the experiment, the speedometer could be seen by the experimenter in the passenger seat, who was to instruct the subject to abort the trial if the speed exceeded 125 km/h (no trials were actually aborted). CVD was continued outside the tunnel in D, and then in a tunnel again in E. F was the deceleration section of the course, indicated by a sign placed at the starting point of F, and G was a U-turn at the end of the test course. As Figure 2B shows, a tarpaulin was laid down in the area of the road designated for the U-turn, and the subjects were instructed to make a U-turn on the tarpaulin and then stop.

The study included daytime and nighttime trials, and the results in sections A, B, D, F, and G were analyzed under conditions of natural light (i.e., no additional light). C and E included artificial light in the tunnel, so they were excluded from the analysis. The average vehicle speed in CVD (section D) was 99.9 ± 10.2 km/h in daytime and 98.0 ± 8.6 km/h at night, showing that the constant vehicle speed was maintained. The average times for driving each section of the course were: acceleration, 17.1 ± 0.3 s in daytime and 17.4 ± 0.3 s at night; CVD, 36.1 ± 0.6 s in daytime and 36.0 ± 0.6 s at night; deceleration, 41.6 ± 1.1 s in daytime and 40.1 ± 1.9 s at night; and U-turn, 28.9 ± 1.0 s in daytime and 31.9 ± 1.0 s at night. There were no significant differences between day and night in any part of the course. While the vehicle was parked, data for 21 s before starting were analyzed.

The subjects performed 1–3 practice drives for the daytime and nighttime trials before putting on the fNIRS probes. The number of practice drives was determined by the subjects themselves, who were asked after each practice drive whether they wanted any more practice. The experimenter accompanied the subject on each practice drive, and practice sessions were added if the CVD velocity was not stable. None of the subjects needed more than three practice drives.

### Experimental equipment and measurement procedures

#### Experimental vehicle

**Figure 3:**
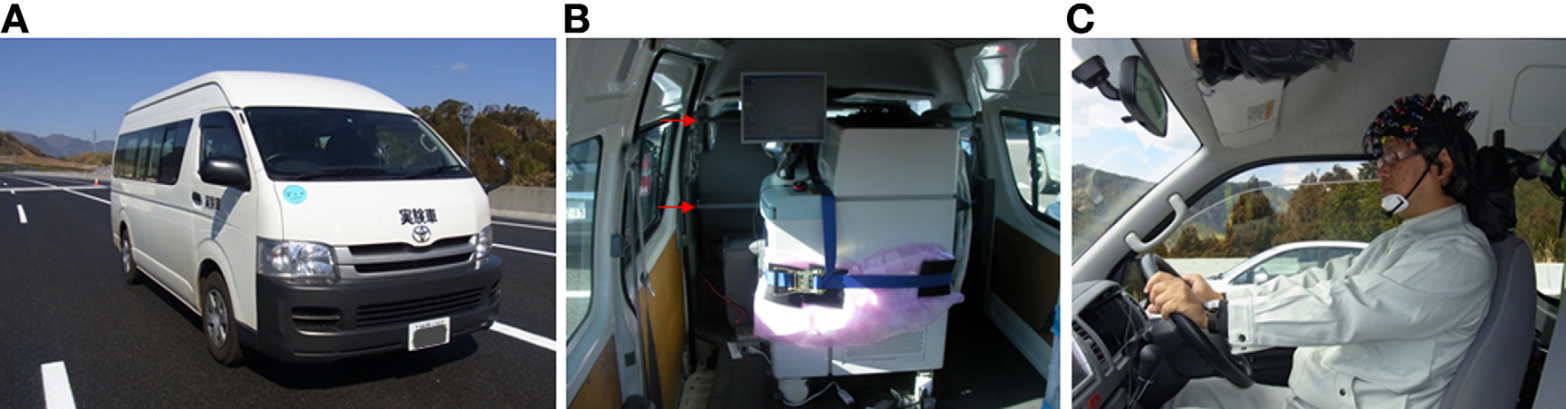
**The experimental vehicle and the fNIRS apparatus. (A)** The experimental vehicle. **(B)** fNIRS equipment mounted in the vehicle. The equipment was secured to two bars attached to the vehicle, indicated by red arrows. **(C)** The driver's seat. A hood covering the subject's head was removed for the photograph. Probes were attached to the subject's head in a way that allowed for moderate changes in driving posture.

A van (“Hiace,” made by the Toyota Motor Corporation; ordinary vehicle classification, with super long, high roof specifications) was used in the experiment (Figure 3A). It is a two-wheel drive, gasoline-powered vehicle with four-speed automatic transmission. A global positioning system receiver and a vehicle speed pulse counter were installed for recording position, speed and acceleration.

#### fNIRS apparatus

A multi-channel fNIRS system (FOIRE-3000, Shimadzu Corporation) was mounted in the vehicle and used to measure hemodynamic responses (Figure 3B). The equipment irradiated three wavelengths of NIR light (780, 805, and 830 nm) to the cerebral cortex, and monitored changes in the hemoglobin (Hb) concentrations. Sampling intervals for measuring changes in the concentration of Hb were set to 70 ms. Conversions from absorbance to Hb concentration changes were performed in the apparatus using the method of Matcher et al. (1995), and measurements were performed in continuous mode. Location-related triggers were entered by an experimenter sitting in the passenger seat, at points indicating the beginning of each section of the course.

The fNIRS device was securely attached to the vehicle using two bars installed behind the driver's seat and a hook on the vehicle floor. The probe line was also attached to the bars behind the driver's seat. Power was supplied to the fNIRS equipment by an DC/AC inverter connected to the battery of the vehicle. To prevent noise due to sunlight, the front and back of the device, where the photomultiplier tubes are located, was covered with black cloth, and the subject also wore a black hood after the probes were attached to the head (Figure 3C).

#### Channel arrangement and registration

**Figure 4:**
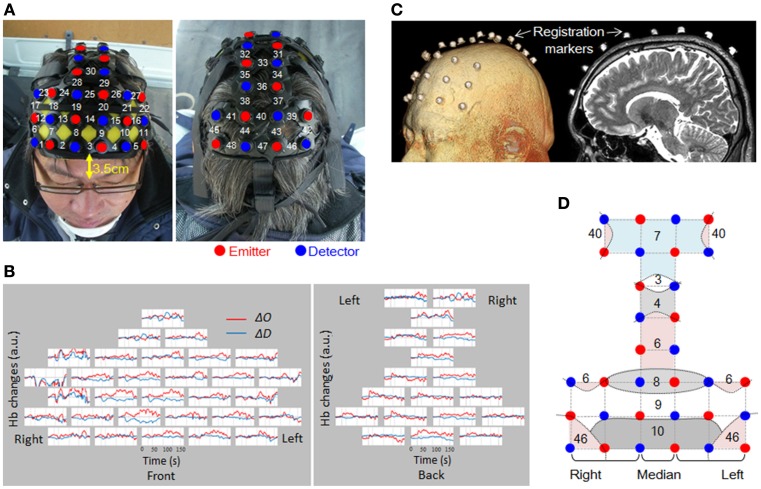
**Channel arrangement and location registration. (A)** Attachment of probes, and channel numbers. **(B)** Raw data from a single trial for one subject. Channels are arranged as shown in **(A)**. **(C)** MRI was used to verify the probe mounting position. **(D)** Straight lines connecting emitters and detectors indicate channel positions. Curved lines show schematic boundaries of the Brodmann areas. Numbers indicate the Brodmann areas as confirmed by MRI. In this study, only the middle column was taken as the median (Ch 3, 14, 25, 30, 33, 36, 40, and 47).

The measurement areas were located on both sides of the prefrontal cortex, motor cortex, and parietal cortex. To ensure the safety of the experiment, we did not measure the occipital lobe. 48 channels were set up using 16 irradiation probes and 16 detection probes (Figure 4A). The distance between the irradiation and detection probes was 3 cm. The head attachment was placed so that the center of the frontmost row was 3.5 cm above the nasion.

When attaching the probes, first, the attachment was mounted on the subject's head, and then the hair was spread out carefully, to expose the skin under each probe. The probes were adjusted one by one so that they pressed the skin surface with a very slight force. The setup was tested to confirmed that there was no interference due to hair or improper contacts, and that the quantity of light received was suitable for data measurement. Figure 4B shows an example of raw data. As shown, the probe settings were carried out with the utmost care to provide the best possible signal-to-noise ratio over all 48 channels. No spiky motion artifacts or artifacts due to vehicle vibration were visually observed.

Positioning of the measurement points was confirmed by MRI with the subject wearing the probe attachment cap fitted with registration markers. MRI was conducted using 3D-T2 weighted images with a 3 Tesla MRI (Philips Co., Achieva 3.0 Quasar Dual 3.0T-MRI). The sampling conditions used the spin-echo method, TE 247 ms, TR 2700 ms, image size 250 × 250 pixels, slice thickness 1.0 mm in the sagittal direction, with an inter-slice gap of 0 mm. The positions of the probes for all the sites measured were confirmed based on the locations of the registration markers (Figure 4C). Figure 4D shows the Brodmann's areas (BA) having the highest correspondence with each channel, from the MRIs of all the subjects. The area measured in the prefrontal cortex covered BA10, BA9, BA8, and BA46. Measurement of BA4 and BA6 in the motor-related areas, and BA3, 7, and 40 in the parietal lobe was also confirmed.

### Analysis

#### Index

We analyzed changes in oxyhemoglobin (ΔO) and deoxyhemoglobin (ΔD) as well as changes in cerebral blood volume (ΔCBV) and cerebral oxygen exchange (ΔCOE), both of which were calculated from ΔO and ΔD. The relationship between ΔO, ΔD, ΔCBV, and ΔCOE is described below based on a secondary square matrix (Yoshino and Kato, 2012).

(1) $$\left(\begin{array}{c} \Delta O+\Delta D\\ -\Delta O+\Delta D \end{array}\right)=\left(\begin{array}{c} 1\\ -1 \end{array}\begin{array}{c} 1\\ 1 \end{array}\right)\left(\begin{array}{c} \Delta O\\ \Delta D \end{array}\right)=\left(\begin{array}{c} \Delta \text{CBV}\\ \Delta \text{COE} \end{array}\right)$$

(2) $$\left(\begin{array}{c} \Delta O\\ \Delta D \end{array}\right)=\frac{1}{2}\left(\begin{array}{c} 1\\ 1 \end{array}\begin{array}{c} -1\\ 1 \end{array}\right)\left(\begin{array}{c} \Delta \text{CBV}\\ \Delta \text{COE} \end{array}\right)$$

ΔCBV (Equation 3) is an index of change in blood volume. ΔCOE (Equation 4) is an index of oxygenation in the blood vessels. A positive value for ΔCOE indicates hypoxic change from ΔCOE = 0, whereas a negative value for ΔCOE indicates hyperoxic change.

(3) $$\Delta CBV=\frac{\left(\Delta D+\Delta O\right)}{\sqrt{2}}$$

(4) $$\Delta COE=\frac{\left(\Delta D-\Delta O\right)}{\sqrt{2}}$$

#### Data processing and statistics

The ΔD and ΔO data were subjected to low-pass filtering at 0.1 Hz to remove any high frequency components. ΔCOE and ΔCBV were calculated using these data. For each of these four indicators, average changes per second for each section of the course (excluding C and E) were determined for each channel. In this process, changes within each section were calculated by first setting the level to zero at the beginning of the section. A total of 35 daytime trials and 36 nighttime trials were analyzed. One daytime trial was excluded because the data did not record correctly.

Analysis of variance with *post-hoc* multiple comparison tests (Scheffe's) were performed to compare average change values for each index for the different sections of the course (at rest, acceleration, constant velocity speed, deceleration and U-turn), for all the sites. These tests were applied separately to the daytime and nighttime data. To investigate differences between the daytime and nighttime experiments, an independent *t*-test was performed for each part of the experiment. The level of significance was set at 5%.

## Results

### Changes in Δ0 and ΔD during actual driving

**Figure 5:**
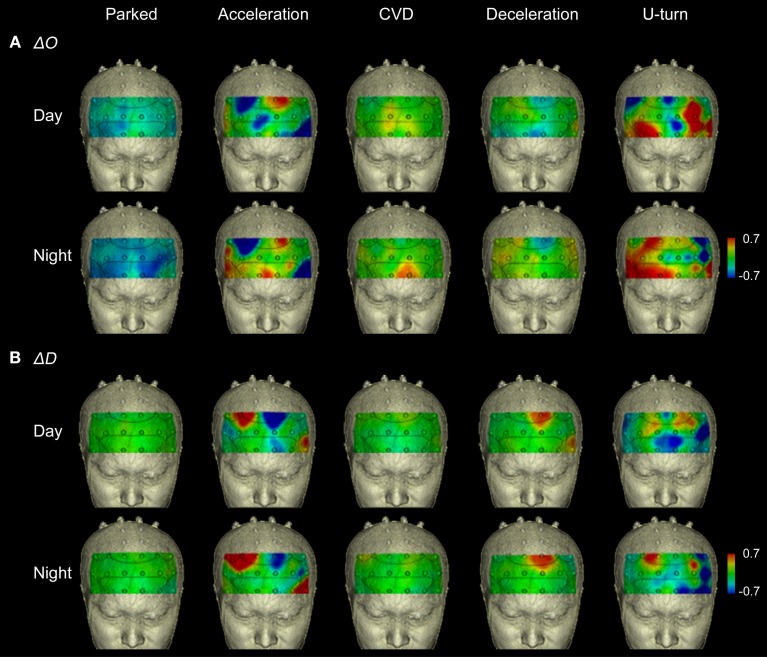
**Functional neuroimages of ΔO and ΔD during actual driving**. Functional imaging of ΔO **(A)** and ΔD **(B)** from the prefrontal cortex (Ch 1-27), during all parts of the experimental task. Dotted lines on the images indicate the boundaries of the Brodmann areas shown in Figure 4D. The images show that increased activation occurred in the same areas in the daytime and nighttime experiments.

Figure 5 shows functional images from the prefrontal cortex for each section of the experiment. They show that the areas activated changed according to the driving operation. The functional imaging results showed high reproducibility in the daytime and nighttime experiments, despite the fact that they were performed on different days.

### Acceleration and deceleration

**Figure 6:**
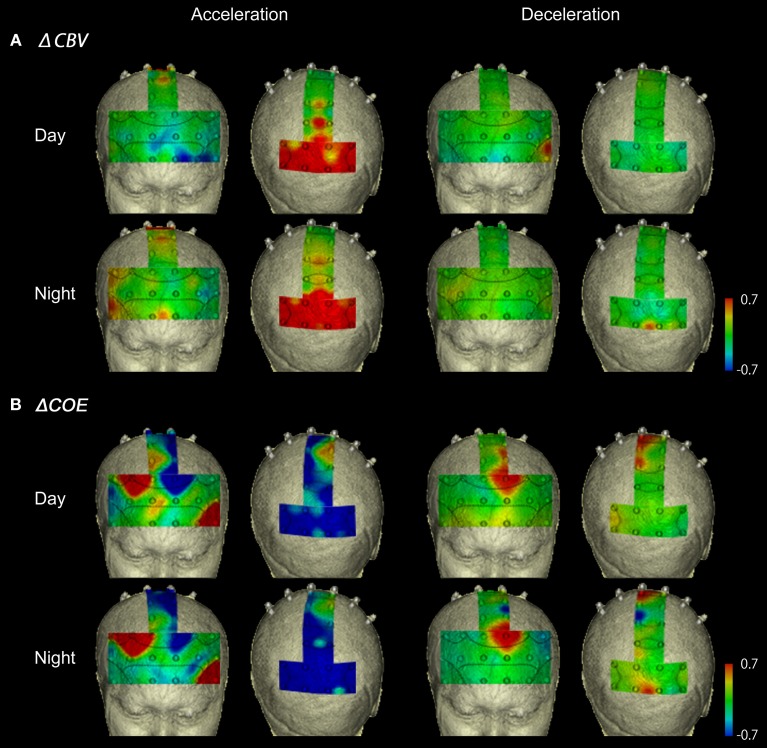
**Functional neuroimages of ΔCBV and ΔCOE from actual driving, during acceleration and deceleration**. Functional imaging of ΔCBV **(A)** and ΔCOE **(B)** from the prefrontal cortex (Ch 1-27), the motor-related areas (Ch 28-36), and the parietal areas (Ch 29-48), during acceleration and deceleration. Note that the prefrontal images and the motor-related and parietal images are reversed on the left and right.

Figure 6A shows functional images of ΔCBV data during acceleration and deceleration. Table A1 (Appendix) shows the results of the comparison of ΔCBV between the parts of the experiment. In the bilateral BA7 and BA40, the medial BA3, and part of the medial BA6 (Ch 33), ΔCBV increased significantly more during acceleration than during deceleration in the daytime. In the nighttime experiment, the significant differences in BA7 and BA40 were reproduced, but the significant differences in BA3 and BA6 disappeared. At night, ΔCBV increased significantly more in the right BA46 and the right BA9 (Ch 17, 23), compared to when the vehicle was parked. There were no sites where ΔCBV increased significantly more during deceleration than during acceleration, either in the daytime or at night.

Figure 6B shows functional images of ΔCOE during acceleration and deceleration. Table A2 (Appendix) shows the results of comparison of ΔCOE between the parts of the experiment. In the daytime, the areas with significant ΔCOE increases during acceleration were the left BA46 and part of the right BA6 (Ch 28). The increase in ΔCOE in the left BA46 was reproduced in the night experiment. In addition, ΔCOE increased significantly at night in part of the right BA6 (Ch 23), part of the right BA9 (Ch 18), and the right BA8. In these same sites, ΔCOE increased in the daytime; these increases were not significant, but indicate reproducibility between the daytime and nighttime experiments during acceleration. In addition, both in the daytime and at night, ΔCOE decreased significantly over a wide range in BA7 during acceleration, compared with other parts of the experiment (Appendix, Tables A2, A3).

In contrast, areas where ΔCOE increased significantly more during deceleration than during acceleration were the medial and the left BA8, the medial and part of the left BA6 (Ch 29, 33), and part of the left BA9 (Ch 20). These significant differences were observed both in the daytime and at night. Also, in the medial BA3 in the daytime, ΔCOE increased more during deceleration than during acceleration (Appendix, Tables A2, A3).

### U-turns and CVD

**Figure 7:**
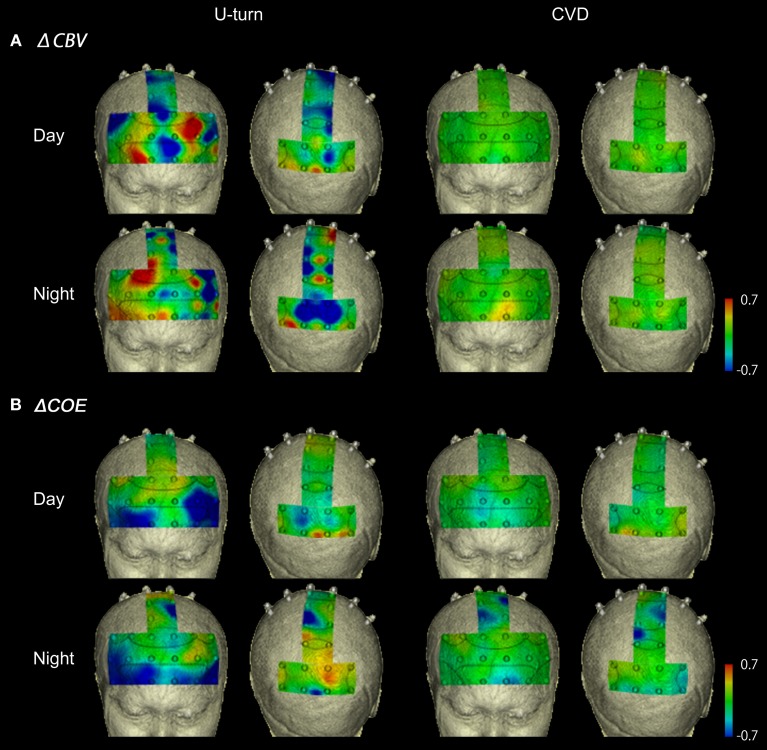
**Functional neuroimages of ΔCBV (A) and ΔCOE (B) during U-turns and CVD**.

Figure 7A shows functional images of ΔCBV data during CVD and U-turns. Table A1 (Appendix) shows the results of the comparison of ΔCBV between the parts of the experiment. In the daytime, ΔCBV increased significantly more during U-turns than during parking in the right BA10 and part of the medial BA7 (Ch 47). At night, the significant difference in the right BA10 was reproduced. ΔCBV also increased significantly more during U-turns than during parking in the right BA46 and part of the left BA10 (Ch 10) at night.

Figure 7B shows functional images of ΔCOE. Table A2 shows the results of comparison of ΔCOE between the parts of the experiment. In the daytime, ΔCOE increased significantly more during U-turns than during acceleration in the prefrontal cortex, in the medial and left BA8, part of the left BA6 (Ch 29), and part of the left BA9 (Ch 20). Among these areas, only the change in the left BA8 was reproduced at night. In the motor-related areas, ΔCOE increased significantly in the medial BA3 in the daytime, and this was reproduced at night. In the nighttime experiment, there was a significant increase in ΔCOE across both sides of BA4. In the parietal lobe, ΔCOE increased significantly in the right BA7 and part of the left BA7 (Ch 48) in the daytime. This response was also spread widely across both sides and the medial BA7 at night (Appendix, Tables A2, A3).

During CVD, there were no areas where ΔCBV increased significantly more than in other parts of the experiment in either the daytime or nighttime experiments. ΔCOE, however, increased significantly more during CVD than during acceleration in the medial, the left BA8, part of the left BA6 (Ch 29), part of BA9 (Ch 20), and part of the right BA7 (Ch 43). Among these areas, the changes in the left BA8, part of the left BA6 (Ch 29), and part of the right BA7 (Ch 43) were reproduced at night.

### Co-occurrence of oxygen responses in the areas measured

The results described in ACCELERATION AND DECELERATION and U-TURNS AND CVD are summarized in Table 1. Increased ΔCOE levels indicate hypoxic changes in blood vessels in the pixels measured. The co-occurrence of these increases in ΔCOE indicate areas recruited during specific driving behaviors.

**Table 1:** **Areas where ΔCOE clearly increased (✓) in each part of the experiment**.

		**Deceleration**		**U-turn**		**CVD**		**Acceleration**	
		**Day**	**Night**	**Day**	**Night**	**Day**	**Night**	**Day**	**Night**
BA8	Right (24)							✓	✓
	Medial (25)	✓	✓	✓		✓			
	Left (26)	✓	✓	✓	✓	✓	✓		
BA7	Right	✓	✓	✓	✓	✓	✓		
	Medial		✓		✓		✓		
	Left	✓	✓	✓	✓	✓	✓		
BA6	Right (23)							✓	✓
	Medial (33)		✓						
	Left (29)	✓	✓	✓		✓	✓		
BA9	Right (18)							✓	✓
	Left (20)	✓	✓	✓		✓	✓		
BA4	Right (34)				✓				
	Left (35)				✓				
BA3	Medial (36)	✓		✓	✓				
BA46	Left							✓	✓
Total		7	8	7	7	6	6	4	4

Numbers in parentheses are channel numbers. Areas without channel numbers recorded increases in more than one channel.

BA8 was involved in all 4 driving operations, and was detected with the highest levels of significance of all the areas measured, followed by BA7, BA6, and BA9. Among the 4 driving operations, deceleration and U-turns activated more brain areas than acceleration and CVD.

### Frontal eye field function during driving

**Figure 8:**
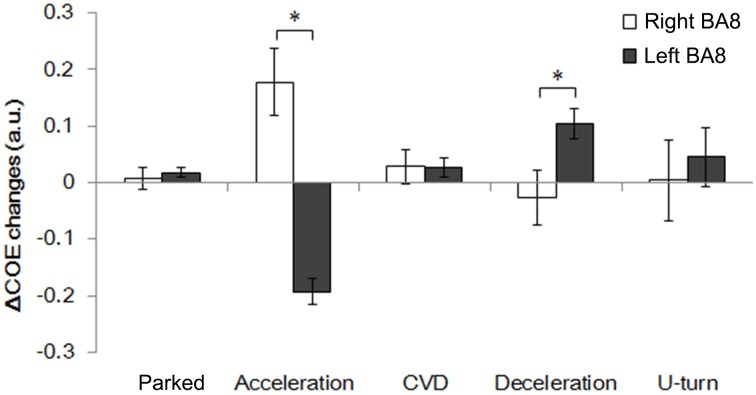
**Comparison of ΔCOE in the left and right BA8**. Changes in ΔCOE in the left BA8 (Ch 24, white) and the right BA8 (Ch 26, black). The graph shows the results of the daytime experiments; the results of the nighttime experiments were similar. (^*^p < 0.05).

Figure 8 shows changes in ΔCOE in both sides of the frontal eye field (FEF, BA8), which showed the most involvement in the driving operations. Differences in laterality were observed during acceleration and deceleration. ΔCOE increased in the right BA8 during acceleration, and in the left BA8 during deceleration (Appendix, Table A4). Reproducibility in BA8 between day and night was high, with no significant differences.

## Discussion

The results of the present study suggest that fNIRS may be an effective technique for evaluating brain activity for the purpose of obtaining objective feedback that can be useful in the construction or improvement of road structures. We were able to measure brain activation during actual driving on an expressway using a multichannel fNIRS system mounted in a vehicle, where we found that the activated areas varied according to the driving operation. A previous fNIRS study investigated actual driving at 30–50 km/h using two channels (NIRO-300) (Harada et al., 2007), but only two sites were measured in the frontal cortex, and localization in the frontal cortex was not clearly shown. The present experiment is the first to image brain functional localization associated with actual expressway driving.

We invested considerable time in preparing the subjects for the experiment and paid particular attention to the safety of the experiment, because we thought that driving while wearing fNIRS probes might induce changes in heart rate and global activation from the autonomic nervous system that would mask localized function related to driving. This did not happen, and the results we obtained showed statistically significant differences in brain functional localization while driving, and showed a high degree of reproducibility in the daytime and nighttime experiments. The ability to perform actual road experiments using fNIRS is potentially advantageous in field studies for intelligent transport systems (ITS). In the future, fNIRS may provide a means for re-evaluating conventional highway design, and creating highways that apply less stress to the brain.

### Cortical activation during actual expressway driving

U-turns and deceleration mobilized more of the brain sites we measured than did acceleration and CVD. During U-turns, there were ΔCOE increases in BA3, BA7, and BA4, and these increases were more robust during nighttime driving. This result may reflect the association between motor function and visual processing required during the manipulation of the foot pedals and the steering wheel (Uchiyama et al., 2012). The significant differences observed in BA3 (the primary somatosensory area) and BA4 (the primary motor area) at night during U-turns were not found in the other parts of the experiment. This may be explained by poor visibility at night for the complex manipulations of the foot pedals and the steering wheel required to back and turn the vehicle when making a U-turn.

A localized increase in ΔCOE and a slight increase in ΔCBV occurred around the medial and the left BA8 during deceleration. This is a situation in which increased blood supply accompanies oxygen metabolism, showing enhanced FEF function. The fact that activation in the parietal lobe increased more during deceleration also suggests that deceleration induces a wider range of brain activation than acceleration.

During acceleration, ΔCOE increased markedly around the right BA8 and in the left BA46 in the prefrontal cortex. The dorsolateral prefrontal cortex, which includes BA46, is considered to be related to attention control during executive functions (Shallice, 1982). In the present study, the dorsolateral prefrontal cortex may have been involved with internal monitoring to reach the target speed within the distance provided. In BA7 (the superior parietal lobule), ΔCOE increased in sections of the course other than acceleration, whereas a global change characterized by a strong decrease in ΔCOE and a strong increase in ΔCBV was observed during acceleration. This response during acceleration, in which only blood volume increased with almost no increase in oxygen metabolism due to neural activity (Yoshino and Kato, 2012), suggests a decline in activation in the superior parietal lobule. Thus, BA8 and BA46 were activated at the same time during acceleration, just as BA8 and BA7 were simultaneously activated in other parts of the experiment. BA46 is involved in the generation of eye movements, and BA7 is involved in visual attention processing that accompanies eye movement (Goldberg et al., 1991). Thus, it can be hypothesized that acceleration requires attention control and generation of eye movements under the motivation of the dorsolateral prefrontal cortex for navigation, while visual attention processing in the superior parietal lobule is reduced. Indeed, in order to reach the target speed of 100 km/h within the short distance of 261 m, the subjects had to depress the accelerator pedal almost to full throttle. In a merging situation on an expressway, possible new traffic safety measures may be based on the assumption that although drivers have high internal control with respect to their goal, their processing of external visual stimuli may be reduced. This suggests that in other parts of the experiment, activity of the superior parietal lobule is increased, by the requirement of greater spatial perception than that needed during acceleration, and by the motor skills required to manipulate the brake, the accelerator, or the steering wheel.

There was no strong activation area detected uniquely in the CVD section of the course, although significant activation around BA8 was detected, as in the rest of the course. There was little need for handling operations on the CVD section of the course, because it was a comfortable road with a brand new surface. We considered the level of brain activation to be low because only minimal movements were required to maintain the car's speed. Thus, when one is simply maintaining vehicle speed on an expressway, one is likely to be driving with low awareness. The above results may be part of the physiological explanation for low awareness driving.

### Frontal eye field functions during expressway driving

The FEF (BA8) is the area that was activated in all parts of the experiment. It is known to be involved in voluntary eye movements, unlike the primary visual cortex in the occipital lobe (Fukushima et al., 2000; Pierrot-Deseilligny et al., 2004). The FEF controls two movements: pursuit eye movements from side to side, and vergence eye movements responsible for depth perception (Gamlin and Yoon, 2000). Vergence is required more in expressway driving, because it is almost straight with unobstructed views, as was this experimental course. It has been reported that the FEF is particularly important during the formation of eye movement signals in three-dimensional space (Fukushima et al., 2002). The detection of increased oxygen metabolism in the FEF suggests that we were able to capture activation associated with voluntary eye movement control in three-dimensional space. Involvement of the occipital and parietal regions in simulation experiments has been reported in several studies (Walter et al., 2001; Horikawa et al., 2005; Spiers and Maguire, 2007; Uchiyama et al., 2012), but there have been few reports of localized activation in the FEF during driving. In a visual simulation experiment without driving operations using fMRI (Graydon et al., 2004), FEF activation was detected in a task that required pushing a button in response to visual cues displayed in a video of actual expressway driving as seen from the driver's seat. The present study resembled that study in that it involved an actual expressway and not artificial road images. We believe our results detected activation of the FEF during three-dimensional processing or high speed visual processing.

Indeed, based on the ΔCOE increases we observed, activation of the FEF was stronger during deceleration and acceleration than during CVD or U-turns. We can hypothesize that the greater the change in speed, the more strongly the FEF is activated. The fact that the FEF was more strongly activated during the deceleration and acceleration parts of the course than during CVD, where there were rapid changes in the field of view at 100 km/h, suggest a relationship between the magnitude of acceleration and the activation of the FEF. In fact, MRI imaging has been reported to show that a professional driver participating in Super GT races at 300 km/h had a bulging FEF (Sumida, 2009). We plan to investigate the relationship between activation of the FEF and the speed of acceleration in a future driving study.

We do not know whether this FEF activity is a response specific to the driver of a vehicle, or whether similar activity occurs in the brain of a person in the passenger seat, and this is an interesting subject. It seems possible that when a person sits the passenger seat and pays the same kind of visual attention as the driver, the same brain activity in the FEF may be detected in the passenger. This kind of control experiment was not performed in this driving experiment, because of the difficulty of reproducing the exact same vehicle behavior, to control the field of vision, with the same subject in the driver's seat and the front passenger seat. This subject may be better approached in a driving simulation study.

ΔCOE increases occurred in the right FEF during acceleration and in the medial and the left FEF during deceleration. Rosano et al. (2002) showed that the right FEF was activated dominantly during saccadic eye movements, although the neural basis of the hemispheric difference remains unknown. Activation of the medial BA8, which includes the supplementary eye fields, was detected together with activation of the left FEF. The lateralization of the FEF, and the connection between the supplementary eye fields and the FEF are novel findings in this study, and will require further consideration in the future.

### Brain functional indices for outdoor experiments

There are many possible effects on the autonomic nervous system in dynamic outdoor experiments, so they require an index (or indices) that can accurately differentiate local activation from global change. When brain function is evaluated based on increases in ΔO, it is difficult to differentiate functional changes from effects of the autonomic nervous system and skin blood flow (Takahashi et al., 2011; Kirilina et al., 2012). Functional localization was poor in an earlier driving simulation experiment using only ΔO (Yanaginuma et al., 2007). In contrast, increases in ΔD can be used to precisely identify sites of neural activity (Ances, 2003). Small ΔD increases such as the initial dip have been difficult to detect, but ΔD increases are likely to occur with higher load tasks (Rupp and Perrey, 2008), and their reproducibility is also high, as we found in the present study.

In recent years, analytical approaches have been proposed that use concentration changes in more than one Hb index to facilitate more precise functional diagnosis (Wylie et al., 2009; Yoshino and Kato, 2012; Sano et al., 2013). ΔCOE, which was used in this study, is similar the subtraction index of ΔO and ΔD that has frequently been used as an index of oxygenation in fNIRS muscle studies. The situation inside the blood vessels as it reflects neural activity has two components, oxygen metabolism and blood volume, and they are both determined by the balance between ΔD and ΔO (Kato, 2004). This means that the use of a single index is likely to give rise to misdiagnosis. In fact, in the present study, ΔCBV increased markedly in the prefrontal cortex during U-turns and in the parietal areas during acceleration, but these increases were not accompanied by increase in ΔCOE. It is highly possible that responses of this kind are merely increases in blood volume, unaccompanied by oxygen metabolism from neural activity. Evaluation of brain functional activation using ΔCOE can be expected to provide new insights in driving studies, which have been previously been performed using BOLD and ΔO, because dynamic signals can be obtained even outdoors. Research that has potential social application, such as in measures for highway safety, urgently requires a reconsideration of indices and analytical methods.

### Further study

The present study clarified some brain regions of interest in the context of traffic safety measures. In particular, it highlighted the importance of the FEF during actual highway driving. There are many visual stimuli on an expressway, such as road signs and lights, and it is possible that devising visual stimuli that would promote or alleviate activation in the FEF could play a part in implementing road safety measures. The study also suggested that the brain functional activation was low during CVD, and this suggests a need for road safety measures for avoiding driving with low awareness. In addition, more areas were activated during deceleration than during acceleration, suggesting that the coordination of more parts of the brain is required for deceleration. In the future, evaluation systems such as that used in this study may provide new suggestions for building safer roads.

Our analysis was focused on the changes during each part of the course, and we did not consider baseline changes over the entire course. There may be differences in baseline levels between indoor simulation experiments and actual driving experiments, when there is likely to be more tension. In the future, it will probably be necessary to compare results of driving simulation tests and actual road tests using the same course and the same indices, to determine the physiological load attributable to actual road driving relative to the baseline level. This type of comparison should elucidate differences between simulation experiments and experiments performed outside the laboratory on actual roads, and help to advance our understanding of brain function in the field of ITS.

Other possible subjects for additional study with larger numbers of subjects include investigation of age and gender differences in brain activity related to driving. There were fewer females than males in this study, not enough to calculate parametric differences and withstand reproducibility, and gender differences were not compared. Also, for the purpose of detecting underlying fundamental data, the subjects were made comfortable with the experimental environment through test runs and practice, and so brain activity when driving in unfamiliar situations was not a part of this study. Because mental tension likely increases on an unfamiliar road, and accidents can be caused by a driver's inability to predict the shape of the road ahead, this is another important subject for investigation.

## Author contributions

Kouji Yamamoto and Hideki Takahashi conceived the intelligent transport systems (ITS) part of the study, and Toshinori Kato designed the neuroimaging part of the study. Kayoko Yoshino performed the experiments and analyzed the data together with Noriyuki Oka; Kouji Yamamoto, and Hideki Takahashi provided valuable help with ITS; and Kayoko Yoshino and Toshinori Kato co-wrote the paper. All the authors discussed the results and commented on the manuscript.

### Conflict of interest statement

The authors declare that the research was conducted in the absence of any commercial or financial relationships that could be construed as a potential conflict of interest.

## Appendix

**Table A1:** **Channels with significant differences in ΔCBV between parts of the task according to a *post-hoc* test**.

	**Part of task (*I*)**	**Part of task (*J*)**	**Brodmann's area**	**Ch**.	**Mean diff. (*I*-*J*)**	***F***	***df***	***P***
**PAIRWISE COMPARISONS**								
Day	Acceleration	Deceleration	BA7, Med.	40	0.118	7.796	4	0.003
				47	0.171	9.383	4	0.003
			BA7, L	41	0.098	2.997	4	0.044
				44	0.230	10.543	4	0.000
				48	0.192	8.479	4	0.000
			BA7, R	39	0.125	11.429	4	0.000
			BA40, L	45	0.120	5.817	4	0.004
			BA40, R	42	0.188	5.717	4	0.003
			BA3, Med.	36	0.115	13.125	4	0.000
			BA6, Med.	33	0.075	10.518	4	0.034
	U-turn	Parked	BA10, R	2	0.164	5.672	4	0.002
				7	0.118	4.204	4	0.004
			BA7, Med.	47	0.137	9.383	4	0.029
Night	Acceleration	Parked	BA46, R	6	0.137	6.436	4	0.001
			BA9, R	17	0.108	3.898	4	0.006
				23	0.103	4.587	4	0.017
		Deceleration	BA7, Med.	40	0.215	11.739	4	0.000
			BA7, R	39	0.092	9.923	4	0.012
			BA7, L	41	0.078	6.006	4	0.038
			BA40, L	45	0.172	7.831	4	0.002
			BA40, R	42	0.129	8.336	4	0.004
	U-turn	Parked	BA10, R	2	0.124	3.054	4	0.026
			BA46, R	1	0.134	3.845	4	0.012
				6	0.117	6.436	4	0.007
			BA10, L	10	0.131	3.231	4	0.017

Ch, channel.

**Table A2:** **Channels with significant differences in ΔCOE between parts of the task in the daytime experiment according to a *post-hoc* test**.

**Part of task (*I*)**	**Part of task (*J*)**	**Brodmann's area**	**Ch**.	**Mean diff. (*I*-*J*)**	***F***	***df***	***P***
**PAIRWISE COMPARISONS**							
Acceleration	Parked	BA7, R	37	−0.193	10.946	4	0.000
			39	−0.149	4.578	4	0.018
			43	−0.200	11.107	4	0.000
	CVD	BA6, R	28	0.096	3.083	4	0.024
		BA7, R	37	−0.153	10.946	4	0.000
			43	−0.163	11.107	4	0.000
	Deceleration	BA7, R	37	−0.175	10.946	4	0.000
			43	−0.188	11.107	4	0.000
	U-turn	BA46, L	5	0.207	3.721	4	0.009
			11	0.293	8.166	4	0.000
		BA7, R	37	−0.112	10.946	4	0.023
			43	−0.122	11.107	4	0.015
CVD	Acceleration	BA8, L	25	0.131	12.902	4	0.001
			26	0.219	14.245	4	0.000
		BA6, L	29	0.141	8.760	4	0.015
		BA9, L	20	0.145	11.693	4	0.002
		BA7, L	48	0.264	7.140	4	0.000
		BA7, R	37	0.153	10.946	4	0.000
			39	0.136	4.578	4	0.044
			43	0.163	11.107	4	0.000
Deceleration	Acceleration	BA8, Med.	25	0.206	12.902	4	0.000
		BA8, L	26	0.298	14.245	4	0.000
		BA6, Med.	33	0.088	3.426	4	0.015
		BA6, L	29	0.218	8.760	4	0.000
		BA9, L	20	0.218	11.693	4	0.000
		BA3, Med.	36	0.086	5.910	4	0.022
		BA7, L	43	0.188	11.107	4	0.035
			48	0.229	7.140	4	0.057
		BA7, R	37	0.175	10.946	4	0.033
			39	0.150	4.578	4	0.043
U-turn	Acceleration	BA8, L	25	0.142	12.902	4	0.000
			26	0.239	14.245	4	0.000
		BA6, L	29	0.172	8.760	4	0.001
		BA9, L	20	0.170	11.693	4	0.000
		BA3, Med.	36	0.099	5.910	4	0.004
		BA7, L	48	0.191	7.140	4	0.025
		BA7, R	37	0.112	10.946	4	0.023
			39	0.141	4.578	4	0.032
			43	0.122	11.107	4	0.015

Ch, channel.

**Table A3:** **Channels with significant differences in ΔCOE between parts of the task in the daytime experiment according to a *post-hoc* test**.

**Part of task (*I*)**	**Part of task (*J*)**	**Brodmann's area**	**Ch**.	**Mean diff. (*I*-*J*)**	***F***	***df***	***P***
**PAIRWISE COMPARISONS**							
Acceleration	Parked	BA46, L	5	0.202	13.818	4	0.000
			11	0.170	12.449	4	0.016
		BA7, R	37	−0.213	20.418	4	0.000
			39	−0.225	6.868	4	0.001
			43	−0.234	21.081	4	0.000
	CVD	BA46, L	5	0.211	13.818	4	0.000
			11	0.190	12.449	4	0.005
		BA8, R	24	0.272	5.372	4	0.012
		BA7, R	37	−0.176	20.418	4	0.000
			43	−0.198	21.081	4	0.000
	Deceleration	BA46, L	5	0.217	13.818	4	0.000
			11	0.207	12.449	4	0.002
		BA6, R	23	0.276	3.728	4	0.029
		BA8, R	24	0.310	5.372	4	0.004
		BA7, R	37	−0.170	20.418	4	0.000
			43	−0.211	21.081	4	0.000
	U-turn	BA46, L	5	0.304	13.818	4	0.000
			11	0.336	12.449	4	0.000
		BA9, R	18	0.290	4.172	4	0.008
		BA6, R	23	0.265	3.728	4	0.032
		BA8, R	24	0.262	5.372	4	0.017
		BA7, R	37	−0.238	20.418	4	0.000
			43	−0.273	21.081	4	0.000
CVD	Acceleration	BA8, L	26	0.161	18.563	4	0.000
		BA6, L	29	0.108	9.560	4	0.015
		BA9, L	20	0.117	11.916	4	0.015
		BA7, Med.	40	0.237	15.536	4	0.000
		BA7, L	38	0.193	7.881	4	0.005
			41	0.234	15.568	4	0.000
			44	0.239	16.298	4	0.000
		BA7, R	37	0.176	20.418	4	0.000
			43	0.198	21.081	4	0.000
Deceleration	Acceleration	BA8, Med.	25	0.142	7.534	4	0.001
		BA8, L	26	0.255	18.563	4	0.000
		BA6, Med.	33	0.129	6.023	4	0.012
		BA6, L	29	0.173	9.560	4	0.000
		BA9, L	20	0.197	11.916	4	0.000
		BA7, Med.	40	0.229	15.536	4	0.000
		BA7, L	38	0.237	7.881	4	0.000
			41	0.235	15.568	4	0.000
			44	0.276	16.298	4	0.000
		BA7, R	37	0.170	20.418	4	0.000
			43	0.211	21.081	4	0.000
U-turn	Acceleration	BA8, L	26	0.101	18.563	4	0.030
		BA3, Med.	36	0.087	3.552	4	0.048
		BA4, L	35	0.243	3.674	4	0.036
		BA4, R	34	0.119	4.756	4	0.007
		BA7, Med.	40	0.282	15.536	4	0.000
		BA7, L	38	0.219	7.881	4	0.001
			41	0.209	15.568	4	0.000
			44	0.243	16.298	4	0.000
		BA7, R	37	0.238	20.418	4	0.000
			39	0.223	6.868	4	0.001
			43	0.273	21.081	4	0.000

Ch, channel.

**Table A4:** **ΔCOE in the left and right FEF**.

	**Part of task**	***t***	***P***
**INDEPENDENT *t*-TEST**			
Right BA8-Left BA8 (daytime)	Parked	−0.467	0.642
	Acceleration	5.965	0.000
	CVD	0.048	0.961
	Deceleration	−2.314	0.024
	U-turn	−0.469	0.641

Differences were significant during acceleration and deceleration.
